# Focal adhesion kinase and paxillin promote migration and adhesion to fibronectin by swine skeletal muscle satellite cells

**DOI:** 10.18632/oncotarget.9010

**Published:** 2016-04-26

**Authors:** Dan Wang, Chun-qi Gao, Rong-qiang Chen, Cheng-long Jin, Hai-chang Li, Hui-chao Yan, Xiu-qi Wang

**Affiliations:** ^1^ College of Animal Science, South China Agricultural University/National Engineering Research Center for Breeding Swine Industry, Guangzhou, Guangdong Province, China; ^2^ Davis Heart and Lung Research Institute, Wexner Medical Center at the Ohio State University, Columbus, OH, USA

**Keywords:** satellite cell, migration, adhesion, focal adhesion kinase, F-actin

## Abstract

The focal adhesion kinase (FAK) signaling pathway contributes to the cell migration and adhesion that is critical for wound healing and regeneration of damaged muscle, but its function in skeletal muscle satellite cells (SCs) is less clear. We compared the migration and adhesion of SCs derived from two species of pig (Lantang and Landrace) *in vitro*, and explored how FAK signaling modulates the two processes. The results showed that Lantang SCs had greater ability to migrate and adhere to fibronection (*P* < 0.05) than Landrace SCs. Compared to Landrace SCs, Lantang SCs expressed many more focal adhesion (FA) sites, which were indicated by the presence of p-paxillin (Tyr118), and exhibited less F-actin reorganization 24 h after seeding onto fibronectin. Levels of p-FAK (Tyr397) and p-paxillin (Tyr118) were greater (*P* < 0.05) in Lantang SCs than Landrace SCs after migration for 24 h. Similarly, Lantang SCs showed much higher levels of p-FAK (Tyr397), p-paxillin (Tyr118) and p-Akt (Ser473) than Landrace SCs 2 h after adhesion. Treatment with the FAK inhibitor PF-573228 (5 or 10 μmol/L) inhibited Lantang SC migration and adhesion to fibronectin (*P* < 0.05), decreased levels of p-paxillin (Tyr118) and p-Akt (Ser473) (*P* < 0.05), and suppressed the formation of FA sites on migrating SCs. Thus FAK appears to play a key role in the regulation of SC migration and adhesion necessary for muscle regeneration.

## INTRODUCTION

Cell migration and adhesion are essential components of many biological processes, such as cell growth, embryonic development, and immune responses and wound healing [1]. As a type of adult stem cell, skeletal muscle satellite cells (SCs) possess a remarkable capacity to repair and regenerate damaged muscle [2]. In response to muscle injury, SCs activate, proliferate, migrate, and then differentiate to form myotubes and replacing existing muscle [3, 4]. A much neglected feature of regeneration is the necessity for SCs to migrate to the injury site to carry out cellular tissue repair. In addition, myogenic differentiation is known to depend on the interaction between SCs and the surrounding extracellular matrix (ECM) during muscle development [5]. Hence, controlled SC migration, facilitated by cell–substrate interactions, is necessary for muscle growth, wound repair and cellular maintenance.

In recent years, the properties of SC migration have been studied extensively. In vertebrate limb development, skeletal muscle SCs migrate from the lateral lip of the dermomyotome into the limb buds, where they differentiate and fuse to form the primary myotubes of limb muscles [6]. Previous studies suggested that multiple growth factors, such as hepatocyte growth factor, fibroblast growth factor type 2 and transforming growth factor β [7–9], are able to accelerate SC migration *in vitro*. Recently, Otto *et al.* found that SC migration was correlated by a blebbing mechanism [10]. Interestingly, this previous study suggested that SCs may make use of lamellipodia and blebbing-based migration depending on the surrounding environment [10], although the regulatory signaling pathway governing these migration behaviors remains unclear.

Focal adhesion kinase (FAK) is a non-receptor tyrosine kinase that is highly overexpressed in hypertrophied skeletal muscle [11]. It is activated through integrin-mediated cell adhesion to the ECM and stimulates the activity of several intracellular signaling pathways, such as the paxillin and phosphatidyl inositol-3 kinase (PI3K) pathways. FAK is involved in the dynamic regulation of focal adhesion (FA) sites, a process that is critical for the control of cell migration and adhesion [12]. Activated FAK binds to cell membrane integrins with the assistance of other proteins, such as paxillin and vinculin, contributing to FA formation, cell adhesion, and cell migration [13, 14]. Considerable evidence indicates that the FAK pathway promotes the migration and adhesion of many types of cells, such as THP-1 monocytes, macrophages, and lung cancer cells [15–17]. Moreover, the activation of FAK is known to promote the growth and differentiation of skeletal muscle cells in culture via the translocation of FAK to costameres [18]. However, the function of FAK signaling in the regulation of SC migration and adhesion has not been addressed experimentally.

Previous studies mainly focused on the proliferation and growth of swine muscle SCs [19, 20]. Information on the regulation of the migration and adhesion of muscle SCs in pigs is scarce, particularly with respect to the mediating role of FAK. Therefore, we hypothesized that the migration and adhesion abilities of SCs isolated from the longissimus dorsi muscles of Lantang (an indigenous pig of China) and Landrace pig were different *in vitro*, then we compared the formation of FAs and F-actin of Lantang SCs and Landrace SCs and analyzed the effects of p-FAK inhibition by PF-573228 on SC migration and adhesion.

## RESULTS

### The migration and adhesion abilities are different in Lantang and Landrace SCs

**Figure 1:**
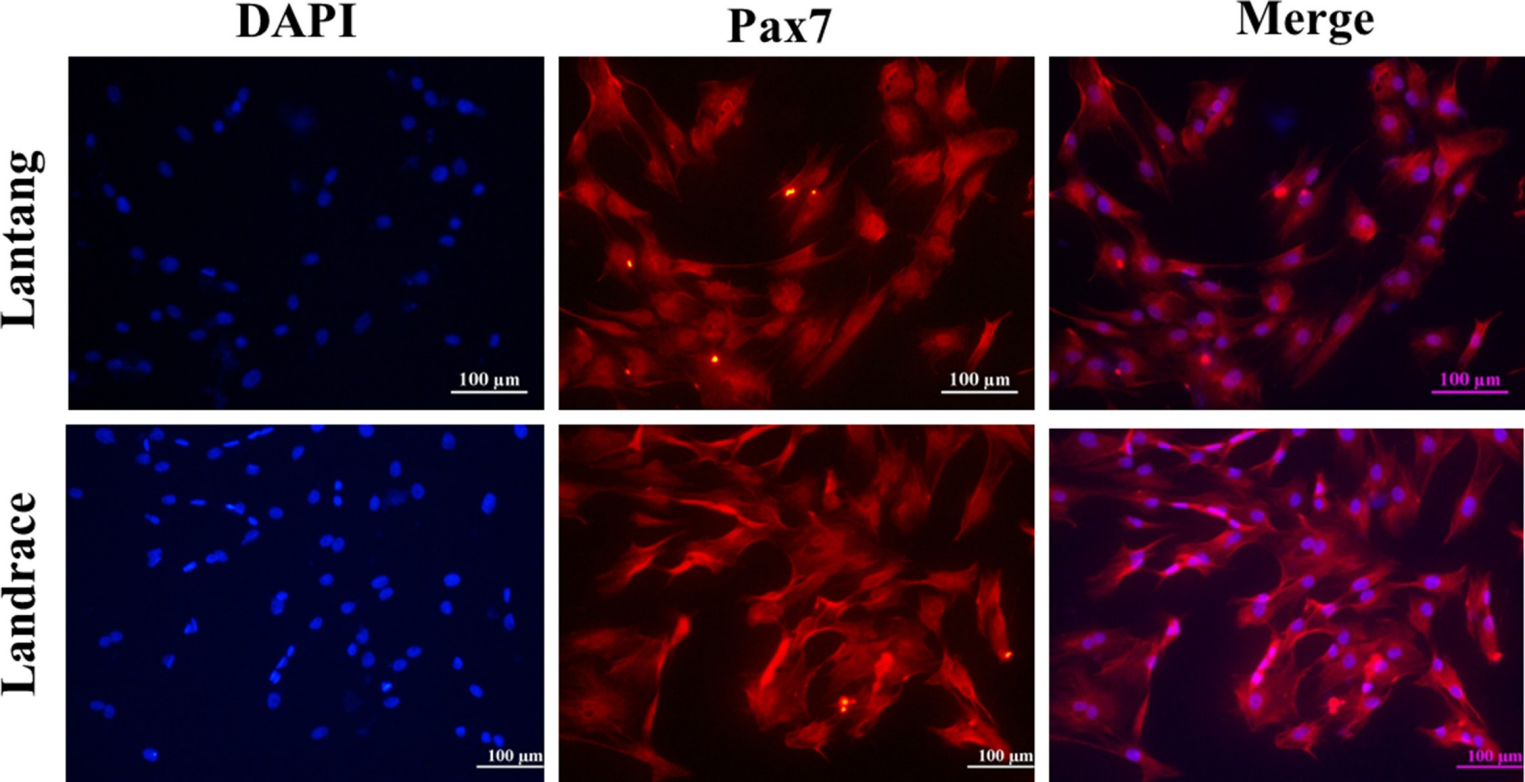
Identification of satellite cells (SCs) by immunofluorescence staining SCs from Lantang and Landrace pigs were seeded in 96-well plates for immunofluorescence. The images represented Lantang and Landrace SCs immunolabelled with anti-human Paired box protein Pax-7 polyclonal antibody, respectively. Pax7 immunostaining is depicted in red, nuclei are counterstained with DAPI (blue). Scale bar: 100 μm.

**Figure 2:**
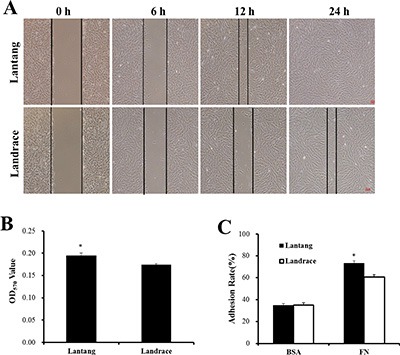
The differences in the migration and adhesion abilities of Lantang and Landrace SCs (**A**) The migration ability of Lantang and Landrace SCs was compared using wound-healing assays. Scale bar: 50 μm. (**B**) Quantification of the number of migrated SCs in the transwell migration assay after 24 h migration. (**C**) Adhesion assays were performed to compare Lantang SCs with Landrace SCs. SCs were seeded in 96-well plates coated with fibronection (FN, 10 μg/mL) or Bovine serum albumin (BSA, 1%). The percent adhesion was calculated by dividing the absorbance of a well by the absorbance of the 100% input control. The results were representative of 3 separate experiments. Bars represent the means ± SEM. *Indicates a significant difference (*P* < 0.05).

Previous studies found that the fiber number and cross-sectional area of the longissimus dorsi muscle were different between Lantang and Landrace pigs [20]. We hypothesized that a difference in SC migration ability is responsible for this phenotype. Firstly, the isolated SCs were identified for the anti-human Paired box protein Pax-7 polyclonal antibody by immunofluorescence analysis. And we found that nearly all of these cells express the transcriptional regulator Pax7 (Figure 1), which marks muscle SCs. Next, the migration abilities of SCs from these pigs were compared using a wound-healing assay on plates coated with FN. Representative images (Figure 2A) showed that SCs migrated into the wound area at 0, 6, 12 and 24 h after wounding. Qualitative results suggested that more Lantang SCs moved into and filled the gap compared with Landrace SCs at 24 h after wounding, implying that Lantang SCs migrated faster than did Landrace SCs (Figure 2A). Cell migration was quantified by examining the number of SCs that migrated from the upper chamber to the lower chamber in a transwell migration assay. The results showed that more Lantang SCs migrated than Landrace SCs did (Figure 2B), which demonstrated that the migration ability of Lantang SCs was superior to that of Landrace SCs.

The adhesion ability of SCs was analyzed using a static adhesion assay, in which FN and BSA coatings were used as positive and negative controls, respectively. After 2 h of adhesion, 73.1% of Lantang SCs adhered to the FN coating, while 60.6% of Lantang SCs were adhered, no significant differences in adhesion to BSA were observed (Figure 2C). These data indicated that the adhesion ability of Lantang SCs was significantly greater than that of Landrace SCs.

### The FAs and F-actin filaments of SCs are distributed at 24 h migration

**Figure 3:**
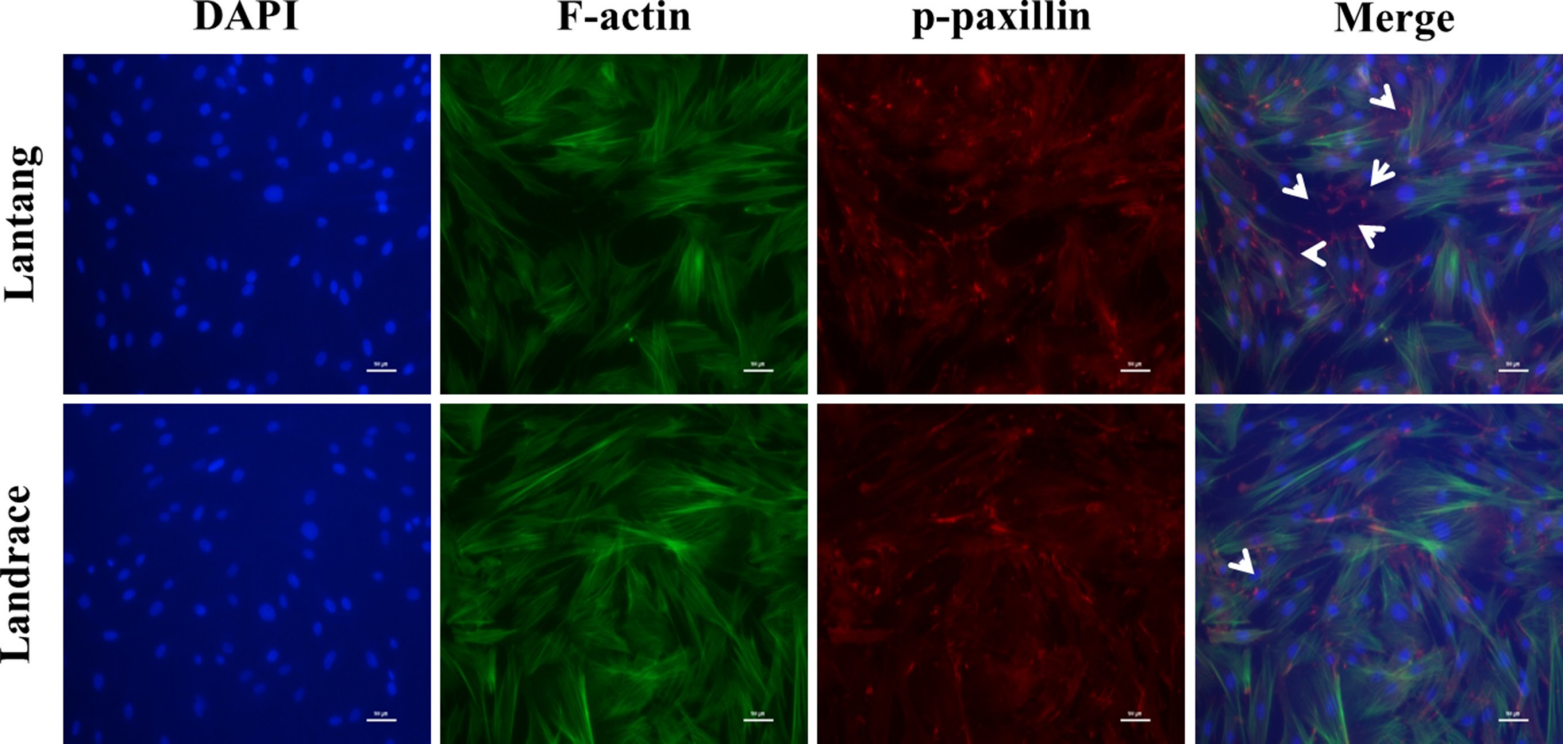
The distribution of focal adhesions (FAs) and F-actin filaments during SC migration grown on FN Representative Immunofluorescence images of nuclear (blue), F-actin filaments (green) and p-paxillin (red) staining in migrating SCs at 24 h are shown. The white arrows indicated polarization of p-paxillin to the leading edge of the migrating SCs. The results were representative of 3 separate experiments. Scale bar: 100 μm.

FAs are a crucial cell adhesion complex and play an essential role in maintaining attachments to the ECM. FAs act as signaling centers from which numerous signaling molecules regulate cell adhesion and migration [21]. Paxillin is a multi-domain adaptor protein that localizes primarily to FA sites. Immunofluorescence staining was used to investigate the phosphorylation level of paxillin (Tyr118) at 24 h after seeding on FN. Compared with Landrace SCs, Lantang SCs expressed many more active FA sites, which were indicated by the presence of p-paxillin, at the edges of the cell (Figure 3). Cell migration and adhesion are coordinated by receptor activation and fast intracellular responses of the actin cytoskeleton. Thus, the F-actin polymerization responses were analyzed in SCs. Representative F-actin staining indicated that Landrace SCs have more extensive F-actin in the cytoplasm than Lantang SCs (Figure 3). Overall, these results suggested that Lantang SCs had more FA proteins and fewer F-actin filaments than Landrace SCs.

### The differential migration and adhesion abilities of SCs are related to FAK signaling pathway

**Figure 4:**
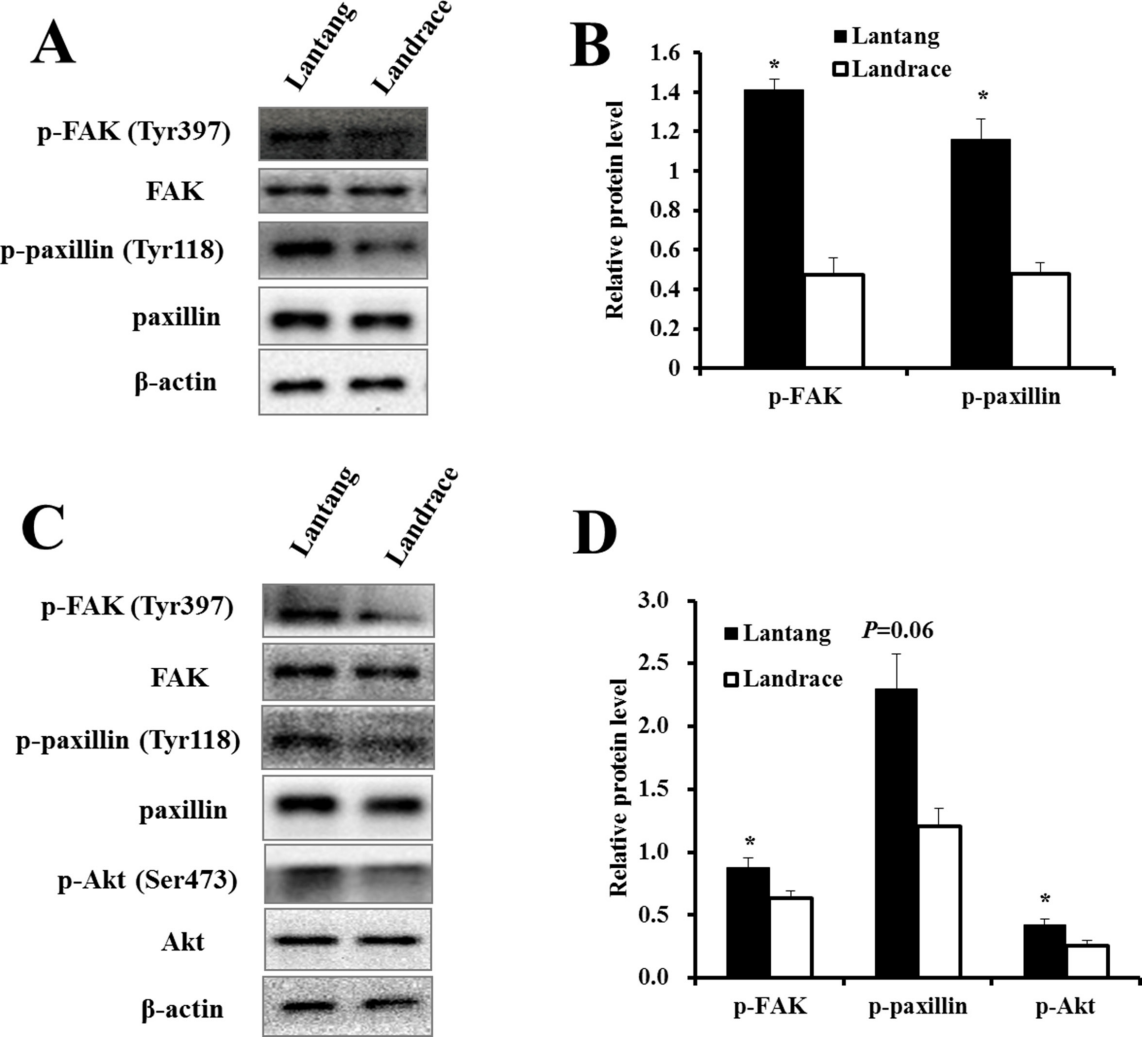
The protein expression of the FAK signaling pathway in the adhesion and migration assays (**A**) Western blot analysis of the phosphorylation state of components of the FAK signaling pathway in SCs after 24 h of migration. (**B**) The phosphorylation levels of FAK and paxillin were presented relative to the respective total protein levels. (**C**) Western blot analysis of the phosphorylation state of components of the FAK signaling pathway in SCs after 2 h of adhesion. (**D**) The phosphorylation fold changes of FAK and Akt were presented as the means, while the level of p-paxillin was presented relative to the β-actin level. β-actin was used as an internal control. The results are confirmed by 3 independent experiments with 5 samples per treatment. The data represent the means ± SEM. *Indicates a significant difference (*P* < 0.05).

We investigated the protein expression of components of the FAK signaling pathway at 2 and 24 h culture in the adhesion and migration assays, respectively. Western blotting analysis showed that the protein expression of p-FAK at Tyr397, a major phosphorylation site during FAK activation, was greater in Lantang SCs compared with Landrace SCs under both conditions (migration and adhesion) (Figure 4A–4C). Additionally, the activation states of paxillin and Akt, which act downstream of FAK, were assessed. The data showed that the protein level of p-paxillin (Tyr118) in Lantang SCs was greater (*P* < 0.05) than that of Landrace SCs during migration (Figure 4B). Lantang SCs presented much greater protein expression of p-paxillin (*P* = 0.06) and p-Akt (Ser473) (*P* < 0.05) relative to Landrace SCs after adhesion for 2 h (Figure 4C–4D). These data indicated that the differences in the migration and adhesion abilities of SCs were associated with the activation of the FAK-paxillin signaling pathway.

### Down-regulation of p-FAK by PF-573228 inhibits SC migration and adhesion

**Figure 5:**
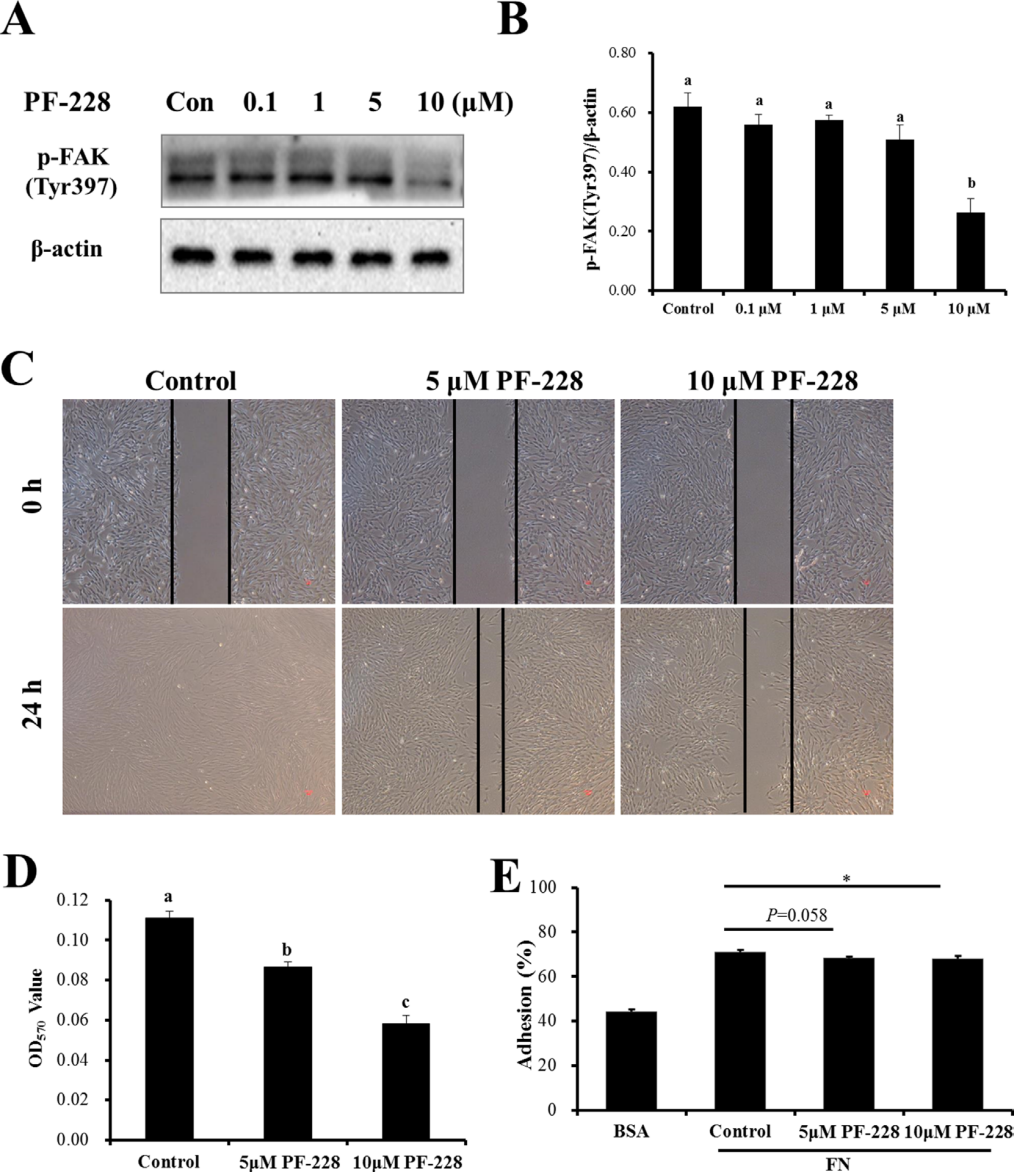
The effect of inhibition of p-FAK by PF-573228 on SC migration and adhesion (**A**) Western blots were performed to assess the inhibition of auto-phosphorylation by the indicated concentrations of the FAK inhibitor, PF-573228 here after referred to as PF-228. Dimethyl sulfoxide (DMSO) was used as a vehicle control. (**B**) The phosphorylation level of FAK was quantified relative to the β-actin level. (**C**) SCs treated with 0, 5, and 10 μmol/L PF-228 were allowed to migrate into the wound area for 24 h after wounding in a wound-healing assay. Representative images are shown. (**D**) Cells treated with 0, 5 and 10 μmol/L PF-228 were allowed to migrate in a transwell for 24 h. Cell migration was quantified using an absorbance assay for the cells that migrated to the lower chamber of the transwell. (**E**) ECM-Cell adhesion assays were used to test the effect of different concentrations of PF-228 on SC adhesion. The results are representative of 3 separate experiments. Bars represent the means ± SEM. *Indicates a significant difference (*P* < 0.05).

To further clarify the FAK signaling pathway responsible for regulating SC migration and adhesion, Lantang SCs were treated with a specific p-FAK inhibitor, PF-573228. The protein expression of p-FAK was detected by Western blot at 24 h after treatment (Figure 5A). The 10 μmol/L dose of PF-573228 was able to inhibit the auto-phosphorylation of FAK (Tyr397) in SCs (Figure 5B). Based on the results, the 5 and 10 μmol/L doses of PF-573228 were used for the subsequent studies. To investigate the effect of PF-573228 on the migration of SCs, a wound-healing assay was performed. Compared with the control group (with DMSO treatment), treatment with PF-573228 at concentrations of 5 and 10 μmol/L caused notably slower wound closure of SCs at 24 h after wounding (Figure 5C); similar results were observed in the transwell migration assay (Figure 5D). After 24 h of PF-573228 treatment, the 10 μmol/L group showed a significant inhibition of cell adhesion ability, as shown by crystal violet staining (*P* < 0.05) (Figure 5E). The percent adhesion was 4.25% (67.9% ± 0.7% vs 71.0% ± 1.1%) decreased in the 10 μmol/L group and a downward trend was observed in the 5 μmol/L group (*P* = 0.058) relative to the control group (Figure 5E). The results showed that down-regulation of p-FAK can significantly reduce the migration and adhesion abilities of SCs.

### The expression of paxillin and Akt was reduced after p-FAK inhibition

**Figure 6:**
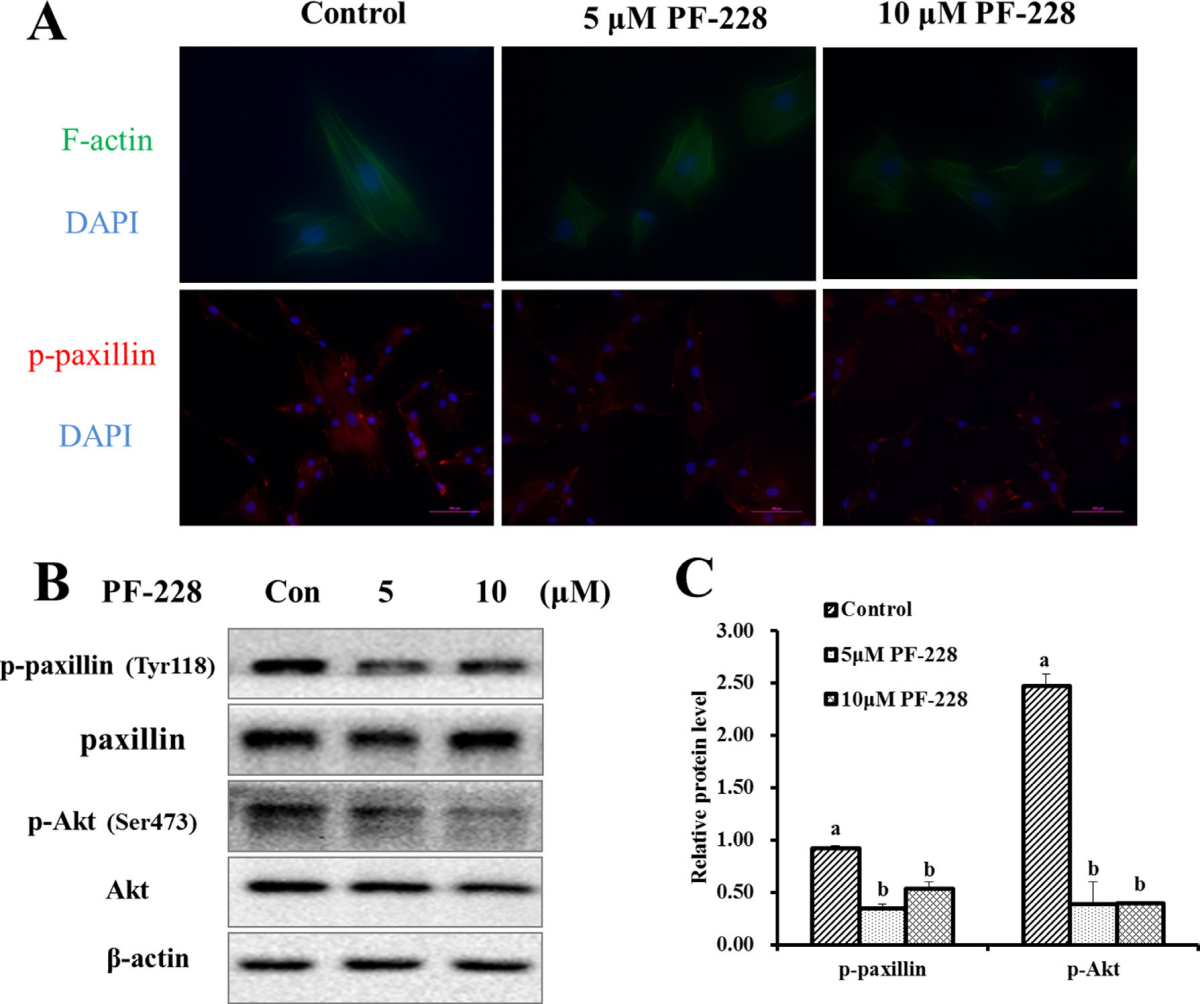
The expression of F-actin filaments, p-paxillin and p-Akt after 24 h of PF-228 treatment (**A**) Immunofluorescence staining was used to examine the changes in F-actin filaments and p-paxillin expression after treatment with PF-228. (**B**) Western blot of p-paxillin, p-Akt in SCs after 24 h of PF-228 treatment. (**C**) The phosphorylation levels of paxillin and Akt were quantified relative to the respective total protein levels. The results shown are representative of 3 independent experiments. Bars represent the means ± SEM, *n* = 3. Scale bar: 100 μm.

To investigate the effect of p-FAK inhibition on the formation of FAs and F-actin filament remodeling, the distribution and localization of the F-actin and p-paxillin were examined after 24 h treatment with PF-573228. Compared with the control group, the 5 and 10 μmol/L PF-573228 groups reduced the number of FA sites, which were indicated by the presence of p-paxillin (Figure 6A). Surprisingly, p-FAK inhibition did not influence on the expression of F-actin filaments (Figure 6A). The protein levels of components of the FAK signaling pathway were also determined after 24 h of PF-573228 treatment. The data showed that the proteins levels of p-paxillin (Tyr118) and p-Akt (Ser473) were significantly down-regulated in the 5 and 10 μmol/L treatment groups when compared with the control group (Figure 6B–6C), respectively. These results suggested that FAK may contribute to SC migration and adhesion via the activation of the downstream effectors paxillin and Akt.

## DISCUSSION

SC migration and adhesion are essential for not only promoting rapid tissue regeneration but also regulating muscle growth. Here, the differences in the migration and adhesion abilities were explored between Lantang and Landrace SCs. The date demonstrates that Lantang SCs have much greater migration and adhesion abilities than Landrace SCs. The reason may due to the differences between Landrace and Lantang SCs in the activation of the FAK-paxillin pathway and the formation speed of FAs. The present study provides the first elucidation that the migration and adhesion abilities of SCs may be used as dynamic factors to improve muscle growth.

Previous studies have shown that the proliferation and growth abilities of Lantang SCs were much greater than those of Landrace SCs [20]. In present study, similar results were observed for the migration and adhesion abilities of SCs. The different migration ability of SCs may be related to growth factors, such as IGF-I, which was shown to be indispensable for regulating SC size in our previous study [19]. Gene microarray results suggested that the mRNA abundance of cell adhesion molecules (ITGB3, PECAM1 and VCAM1) is significantly different between Lantang SCs and Landrace SCs (data not shown). We hypothesized that this difference in the expression of CAMs would affect the adhesion abilities of SCs from Lantang and Landrace pigs, which is proved to be correct.

FAK is a FA-associated protein kinase involved in cellular spreading and migration [22]. Its tyrosine phosphorylation plays an indispensible part in the signal transduction and formation of FAs, which are formed at the ECM-integrin junction [23, 24]. In the current study, the FAK-paxillin signaling pathway was found to be activated during SC migration and adhesion. Inhibition of p-FAK activity significantly suppressed the migration and adhesion of SCs, which clearly demonstrates that FAK is a key regulator of SC biology, promoting not only cell migration but also cell adhesion. In addition, FAK activity is also stimulated by upstream molecules, such as integrins, which are a family of transmembrane heterodimeric glycoprotein receptors that physically link the ECM to the intracellular cytoskeleton at FAs. It has been widely demonstrated that FN stimulates cell migration through the activation of integrin β1 and β3 [25]. However, our results showed the mRNA abundances of integrin β1, integrin β3 and FN were not significantly different between Lantang and Landrace SCs (data not shown). While the main reason for this difference is that the function of integrin β1 and β3 on cell migration may depend mainly on the different cell species, the underlying regulative mechanisms in the pig skeletal muscle SC are worth to depth explore.

Multiple downstream molecules of FAK, including paxillin and Akt, have been identified that mediate FAK regulation of migration and adhesion in various cells [26, 27]. In this study, inhibition of p-FAK (Tyr397) also decreased the protein level of p-paxillin (Tyr118), which is closely related to FAK activity. Akt is a central mediator of FAK-PI3K signaling and regulates cell migration and invasion through the modulation of the actin cytoskeleton and the assembly of integrin adhesion complexes [26]. Thus, the expression of p-Akt (Ser473) was detected to analyze the inhibitory effect of p-FAK on the activation of Akt. Inhibition of p-FAK reduced the activating phosphorylation of Akt, suggesting a possible role for phosphorylation in FAK-dependent Akt signaling. In brief, these results demonstrate that FAK has a positive effect on migration and adhesion in pig muscle SCs.

Cell migration is a coordinated process that involves rapid changes in actin filament dynamics together with the formation and disassembly of FA sites [28]. In other words, integrin interactions with the ECM readily form FAs that can be attached to the actin cytoskeleton for structural support and can engage in intracellular signaling that can drive cell migration [29, 30]. In this study, we found that Lantang SCs formed more FA sites, indicted by p-paxillin, but less F-actin than Landrace SCs and that the FAK-paxillin signaling pathway takes part in this process. FAK is a primary signaling mediator of dynamic changes in F-actin cytoskeletal reorganization. The ability of cells to adhere is primarily depend on the number of FA sites which is different between Lantang and Landrace SCs. F-actin participates in FA dynamics and modulates the direction of lamellipodium protrusion [31, 32], consequently controlling the velocity and directionality of cell migration. In the present study, Landrace SC had less migration ability, and expressed more abundant F-actin than Lantang SC. Additionally, Mizutani *et al.* found that excess F-actin causes a decrease in cell plasticity, thus inhibiting cell migration [33]. These results indicate that the negative regulation of F-actin on cell migration in pig skeletal muscle SC.

PF-573228 is a p-FAK-specific inhibitor, which interacts with FAK in the ATP-binding pocket and effectively blocks the catalytic activity of recombinant FAK protein in a variety of normal cell lines [34]. In the present study, treatment of SCs with 10 μmol/L PF-573228 inhibited FAK phosphorylation on Tyr397, decreased the protein levels of p-paxillin and p-Akt, and reduced the number of FAs. Similar findings have been reported by Slack-Davis *et al.*, who observed that treatment with PF-573228 suppressed FN-induced migration and concomitant with the inhibition of FA turnover [34]. The observation that PF-573228 inhibited cell migration and adhesion in a dose-dependent manner supports a role for FAK activity in the regulation of cell migration and adhesion.

In conclusion, the different migration and adhesion abilities of Lantang and Landrace SCs are associated with differences in the distribution and localization of F-actin and FAs, which are regulated by the activation of the FAK-paxillin signaling pathway. The results of this study may provide potential molecular targets for the regeneration of muscle and the treatment of muscular dystrophies. However, further studies are required to determine whether this signaling pathway can modulate the regeneration of skeletal muscle in addition to promoting migration and adhesion. Additionally, more in-depth experiments also need to be performed *in vivo*.

## MATERIALS AND METHODS

### Animals

Lantang or Landrace piglets at 1-day-old were used and handled in accordance with the directives of the Institutional Animal Care and Use Committee of the South China Agricultural University (Guangzhou, China).

### Primary pig skeletal muscle SC culture

We followed the pig skeletal muscle SC separation method described by Wang *et al.* [20]. Briefly, we obtained longissimus dorsi muscle tissue from 1-day-old Lantang or Landrace pigs (2–3 g) using 0.2% collagenase Type II (Sigma, St. Louis, MO, USA) in a 2 h enzyme digestion method to release the SCs. Subsequently, the pig SCs were purified using the differential adhesion method described by Gharaibeh *et al.* [35]. Cells were cultured in DMEM/F-12 (Gibco, Grand Island, NY, USA) supplemented with 10% fetal bovine serum (FBS; Gibco, Carlsbad, CA, USA) at 37°C and 5% CO_2_ in a standard cell culture incubator. Immunocytochemistry analysis showed that the isolated SCs were positive for the Rabbit anti-human Paired box protein Pax-7 polyclonal antibody (Wuhan Huamei Biotech Co. Ltd, Wuhan, China).

### Cell-migration assays

### Wound-healing assay

SCs were seeded at a density of 5 × 10^5^ cells/well in a FN-coated 6-well culture plate (Corning, NY, USA). After reaching 80–85% confluence, the confluent monolayer was then scratched with a 200 μL pipet tip and washed twice with phosphate buffered saline (PBS). The cells were then cultured with DMEM/F-12 supplemented with 1% FBS to minimize the effect of cell proliferation. For inhibition experiments, the wells were filled with 2 mL of serum-free DMEM/F-12 with 0 (control), 5 and 10 μmol/L FAK inhibitor PF-573228 (Selleck Chemical, Houston, TX, USA) for 24 h at 37°C. Images of the wounds were acquired at 0 and 24 h using a microscope (NIS-Elements, Nikon, Japan).

### Transwell migration assay

For this assay, 24-well transwell culture plates (#3422) were obtained from Corning(NY, USA). DMEM (600 μL) containing 20% FBS was added to the lower chambers. SCs suspended in 100 μL DMEM with 1% FBS were added to the upper chamber of the transwell unit. The cells were allowed to migrate for 24 h at 37°C in a humidified atmosphere containing 5% CO_2_. Afterwards, the medium (containing non-migrated cells) from the upper chamber was carefully removed by aspiration. The cells from the underside of the transwell inserts were fixed with 4% paraformaldehyde (PFA) for 30 min, stained with 0.1% crystal violet for 20 min, and dissolved in 33% acetic acid. The absorbance of the reaction product was measured with a microplate reader (Bio-Rad, CA, USA) at a wavelength of 570 nm.

### Cell adhesion assays

Ninety-six-well plates were coated with 10 μg/mL FN (BD Bioscience, Bedford, MA, USA) and 1% bovine serum albumin (BSA) overnight at 4°C and then blocked with 1% BSA for 30 min at 37°C. SCs were harvested using trypsin/EDTA (Gibco, Carlsbad, USA), suspended in serum-free medium, added to the coated plates at 1 × 10^4^cells/well, and allowed to adhere for 120 min at 37°C. For inhibition experiments, SCs were treated with 0, 5 and 10 μmol/L PF-573228 and incubated for 24 h prior to the adhesion experiments. Subsequently, the non-adherent cells were removed by washing the cells two times with PBS. The adherent cells were fixed in 4% PFA, stained using 0.1% crystal violet for 30 min at room temperature, and then dissolved in 70% ethanol for 2 h. The absorbance at 570 nm was measured using a microplate reader. The results were expressed as percent adhesion relative to the number of initial seeded cells, which represented 100% attachment.

### Immunofluorescence staining

SCs were grown on FN-coated plates and then fixed with 4% PFA for 15 min, followed by permeabilization with 0.1% Triton-X-100 in PBS for 10 min. After the cells were blocked with 1% BSA plus 10% normal serum for 30 min, F-actin was detected by staining with FITC-phalloidin (Sigma-Aldrich, St. Louis, MO, USA) for 30 min at 22–25°C. SCs were stained with the primary antibody anti-phospho-paxillin (Cell Signaling technology, Beverly, MA, USA) for 90 min and then probed with the goat anti-rabbit IgG (Bioworld Technology, Louis Park, MN, USA). The nuclei were labeled with 4′,6-diamidino-2-phenylindole (DAPI) for 5 min at room temperature and then washed three times with PBS. Images were obtained using an Immunofluorescence microscope.

### Western blot assay

SCs were washed twice with cold PBS and incubated in a lysis buffer (RIPA, BioTeke, Beijing, China) containing 1 mmol/L protease inhibitor PMSF (Sigma, St. Louis, MO, USA) and phosphatase inhibitors (Invitrogen, Carlsbad, CA, USA) on ice, followed by centrifugation at 12,000 g and 4°C for 15 min. Protein concentration was determined using a Micro BCA Protein Assay Kit (Thermo Fisher Scientific, Rockford, USA). Protein samples (15 μg) were loaded onto a 10% polyacrylamide gel and transferred onto PVDF membranes using semidry methodology, as previously described [36]. Membranes were probed using specific antibodies: anti-FAK (#3285), anti-phospho-FAK (Tyr397; #8556), anti-paxillin (#3283), anti-phospho-paxillin (Tyr118; #2541), anti-Akt (#9272) or anti-phospho-Akt (Ser473; #9271), which were purchased from Cell Signaling Technology (Beverly, MA, USA). Anti-β-actin (AP0060), anti-rabbit IgG (BS13278) and anti-mouse IgG (BS30503) were obtained from Bioworld Technology (Louis Park, MN, USA). The proteins were visualized with the ECL-Plus Western blotting reagent in a FluorChem M system (Cell BioSciences, San Leandro, CA, USA). Band density was analyzed by Image J (http://rsb.info.nih.gov/ij/).

### Statistics

Data are expressed as the mean ± SEM. The results were analyzed with Student's *t*-test and One-Way ANOVA using SAS (version 9.2; SAS Inst. Inc., Cary, NC) software. Differences among groups were determined using Duncan's multiple-range test. The differences between treatments were considered statistically significant at *P* < 0.05, the difference trend was defined at 0.05 < *P* < 0.1.
